# 4-(Phenylsulfanyl)butan-2-One Suppresses Melanin Synthesis and Melanosome Maturation *In Vitro* and *In Vivo*

**DOI:** 10.3390/ijms160920240

**Published:** 2015-08-26

**Authors:** Shing-Yi Sean Wu, Hui-Min David Wang, Yi-Shan Wen, Wangta Liu, Pin-Hui Li, Chien-Chih Chiu, Pei-Chin Chen, Chiung-Yao Huang, Jyh-Horng Sheu, Zhi-Hong Wen

**Affiliations:** 1 Department of Marine Biotechnology and Resources, National Sun Yat-Sen University, Kaohsiung 804, Taiwan, ROC; E-Mails: sunbear@cm1.hinet.net (S.-Y.S.W.); davidw@kmu.edu.tw (H.-M.D.W.); weneasa@gmail.com (Y.-S.W.); betty8575@yahoo.com.tw (C.-Y.H.); 2 Department of Fragrance and Cosmetic Science, Kaohsiung Medical University, Kaohsiung 807, Taiwan, ROC; E-Mail: s27159@gmail.com; 3 Graduate Institute of Natural Products, Kaohsiung Medical University, Kaohsiung 807, Taiwan, ROC; 4 Center for Stem Cell Research, Kaohsiung Medical University, Kaohsiung 807, Taiwan, ROC; 5 Department of Biotechnology, Kaohsiung Medical University, Kaohsiung 807, Taiwan, ROC; E-Mails: liuwangta@kmu.edu.tw (W.L.); cchiu@kmu.edu.tw (C.-C.C.); 6 Center for Infectious Disease and Cancer Research, Kaohsiung Medical University, Kaohsiung 807, Taiwan, ROC; 7 Department of Biological Sciences, National Sun Yat-sen University, Kaohsiung 804, Taiwan, ROC; 8 Translational Research Center, Cancer Center, Department of Medical Research, and Department of Obstetrics and Gynecology, Kaohsiung Medical University Hospital, Kaohsiung Medical University, Kaohsiung 807, Taiwan, ROC; 9 Doctoral Degree Program in Marine Biotechnology, National Sun Yat-sen University and Academia Sinica, Kaohsiung 804, Taiwan, ROC; E-Mail: peichin1128@gmail.com; 10 Department of Medical research, China Medical University Hospital, China Medical University, Taichung 404, Taiwan, ROC

**Keywords:** 4-(phenylsulfanyl)butan-2-one, melanogenesis, tyrosinase, melanosome maturation, zebrafish

## Abstract

In this study, we screened compounds with skin whitening properties and favorable safety profiles from a series of marine related natural products, which were isolated from Formosan soft coral *Cladiella australis*. Our results indicated that 4-(phenylsulfanyl)butan-2-one could successfully inhibit pigment generation processes in mushroom tyrosinase platform assay, probably through the suppression of tyrosinase activity to be a non-competitive inhibitor of tyrosinase. In cell-based viability examinations, it demonstrated low cytotoxicity on melanoma cells and other normal human cells. It exhibited stronger inhibitions of melanin production and tyrosinase activity than arbutin or 1-phenyl-2-thiourea (PTU). Also, we discovered that 4-(phenylsulfanyl)butan-2-one reduces the protein expressions of melanin synthesis-related proteins, including the microphthalmia-associated transcription factor (MITF), tyrosinase-related protein-1 (Trp-1), dopachrome tautomerase (DCT, Trp-2), and glycoprotein 100 (GP100). In an *in vivo* zebrafish model, it presented a remarkable suppression in melanogenesis after 48 h. In summary, our *in vitro* and *in vivo* biological assays showed that 4-(phenylsulfanyl)butan-2-one possesses anti-melanogenic properties that are significant in medical cosmetology.

## 1. Introduction

Manufacturers claim that many of their cosmeceutical ingredients from natural products and chemically synthesized compounds have bio-functional properties. Marine natural resources have become a new inspiration of drug development research in recent years. Our group discovered and isolated several pure compounds from soft corals, which exhibit neuroprotective and anti-inflammatory activities [1,2,3,4,5]. According to previous research, two chemical constituents, austrasulfone and dihydroaustrasulfone alcohol, of Formosan soft coral *Cladiella australis* show significant *in vitro* anti-inflammatory activity [6]. In this study, we continued to screen compounds with skin whitening properties and favorable safety profiles from a series of sulfur-containing compounds that are structurally related to the marine natural product, austrasulfone, which has a four-carbon carbonyl group and an unsaturated phenyl group (similar to the vinyl group for sp^2^ hybridization) attached to sulfur. 4-(phenylsulfanyl)butan-2-one was found to be a safer and more effective ingredient than traditionally used agents for cosmetic and medical applications.

In the cosmetic business, skin whitening is one of the most popular demands in Asia. Skin color is affected by melanin, which is produced in melanocytes in the basal layer of human epidermis. Melanin is formed through the activity of several melanogenesis-related proteins, such as microphthalmia-associated transcription factor (MITF), tyrosinase, dopachrome tautomerase (DCT, Trp-2), tyrosinase-related protein-1 (Trp-1), and glycoprotein 100 (Gp100) [7,8]. MITF is currently believed to regulate melanocyte pigmentation, proliferation, and survival [9,10]. Tyrosinase, Trp-2, and Trp-1 are three main catalytic enzymes in melanin synthesis [11], and Gp100 is involved in melanosome maturation. In addition, MITF has been shown to effectively transactivate tyrosinase, Trp-1, and Trp-2 melanogenic genes *in vitro* and *in vivo* [12,13]. Thus, suppression of these melanogenesis-related proteins is a major strategy for anti-melanogenesis [14,15,16].

Zebrafish models have become a highly advantageous vertebrate animal system for high-throughput screening of melanogenic regulatory agents because zebrafish are small in size, inexpensive, easy to handle, short-lived, and have transparent embryos and physiological similarity to mammals [17]. The zebrafish pigment pattern model provides the opportunity to study melanocyte development and melanin genesis. Moreover, safety pharmacology screenings, biochemical stability testing, and *in vivo* cytotoxic performance testing can be done through this embryonic sensitivity evaluation method.

In this work, we identified a sulfur-containing compound, 4-(phenylsulfanyl)butan-2-one, as a promising and potential skin whitening agent. In the mushroom model, it acted as a non-competitive tyrosinase inhibitor. In murine melanoma B16-F10 cells, we demonstrated that it suppressed melanin production, reduced the protein expression of melanosome maturation related proteins, and inhibited tyrosinase activity. Finally, in zebrafish we illustrated that it repressed *in vivo* pigmentation reversibly, and it maintained satisfactory treatment survival rates. Thus, this target compound could have significant application in the cosmetic field.

## 2. Results

### 2.1. Assay on Mushroom Tyrosinase Inhibition

**Figure 1:**
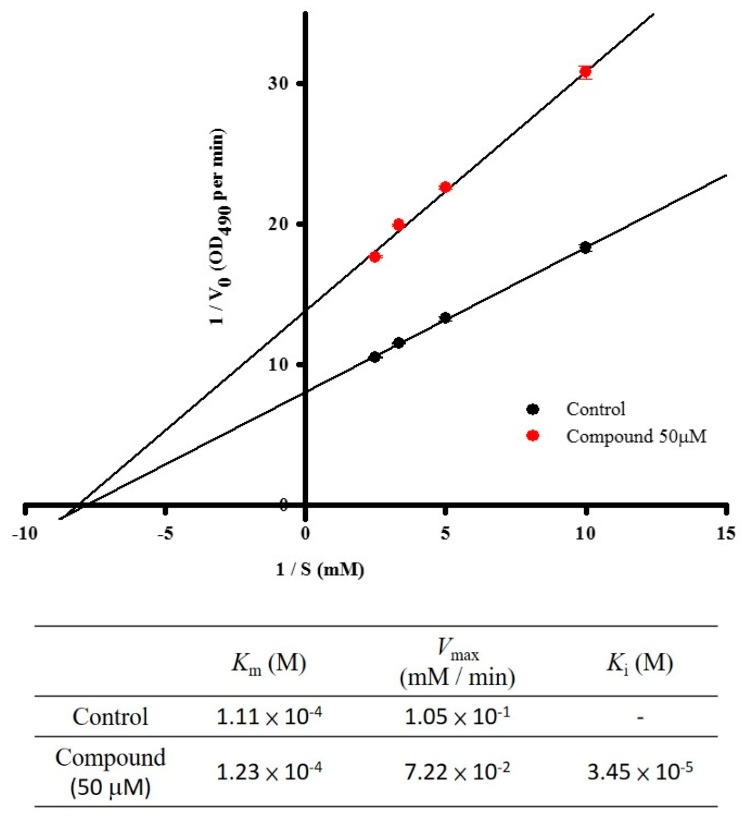
The inhibitory mechanism of 4-(phenylsulfanyl)butan-2-one on mushroom tyrosinase. The data for Lineweaver-Burk plots were obtained as mean values of three independent assays with various concentrations of l-tyrosine as the substrate. The reactions were performed in the presence of 4-(phenylsulfanyl)butan-2-one at 50 µM.

We calculated the mushroom tyrosinase inhibition activity of dozens of pure compounds from marine creatures *in vitro*. We searched for a new and efficient substance for hyper-pigmentation prevention and skin whitening and discovered that one compound, 4-(phenylsulfanyl)butan-2-one, significantly diminished mushroom tyrosinase activity (Table 1), thus, we then synthesized it. It exhibited strong dose-dependent inhibitions of mushroom tyrosinase activities. Additionally, we verified the tyrosinase enzyme kinetics with 4-(phenylsulfanyl)butan-2-one at various concentrations of the l-tyrosine substrate (0.1, 0.2, 0.3, and 0.4 mM). The Lineweaver–Burk equation plot (Figure 1) confirmed that it acts as a noncompetitive inhibitor. The *K*_m_ value was 1.11 × 10^−4^ M, and the *V*_max_ value of mushroom tyrosinase activity was 1.05 × 10^−1^ mM/min, respectively, with no inhibitor. The kinetic analysis in the presence of 50 µM testing compound revealed a *K*_m_ value of 1.23 × 10^−4^ M, *V*_max_ value of 7.22 × 10^−2^ mM/min, and *K*_i_ value of 3.45 × 10^−5^ M.

**Table 1:** The inhibitory effect of 4-(phenylsulfanyl)butan-2-one on mushroom tyrosinase. Data were expressed as a mean value of three independent experiments. Arbutin (100 µM) and PTU (100 µM) were used as positive controls for this assay.

Compounds	(µM)	Tyrosinase Inhibition (%)
Control	0	0.00
4-(Phenylsulfanyl)butan-2-one	10	17.22 ± 1.29
	50	48.14 ± 1.48
	100	82.32 ± 2.56
Arbutin	100	83.44 ± 1.65
PTU	100	95.27 ± 2.85

### 2.2. Cytotoxicity of 4-(Phenylsulfanyl)butan-2-one on Multiple Cells

**Figure 2:**
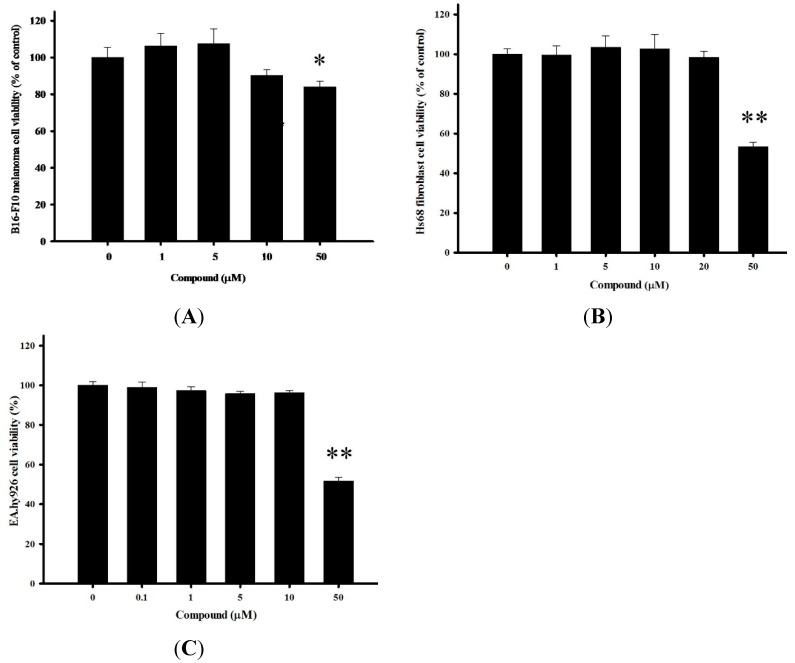
Cell viabilities of (**A**) B16-F10, (**B**) Hs68, and (**C**) EA.hy926 cells after treatments with various concentrations of 4-(phenylsulfanyl)butan-2-one for 24 h (* *p* < 0.01 and ** *p* < 0.001, compared with the concentration at 0 µM).

Preliminarily data from a number of *in vitro* mushroom tyrosinase assay screenings with pure marine constituents and chemically synthesized substances were used to identify 4-(phenylsulfanyl)butan-2-one for further studies. Initially, we measured mouse dermal melanoma B16-F10 cell viability via the Alamar Blue assay to investigate whether our compound causes cytotoxic damage (Figure 2A). To be a promising skin-lightening agent, the compound should have nontoxic side effects, and should be harmless, without undesirable damage. The testing sample was incubated 48 h at suitable concentrations ranging from 1 to 50 µM to examine dose-dependent properties. At 4-(phenylsulfanyl)butan-2-one concentrations lower than 10 µM, cell viability was over 90%, and at the highest dose (50 µM), viability was still higher than 80%. In Figure 2B,C, we also examined cytotoxicity in two other normal human cells, human foreskin fibroblasts (Hs68) and human umbilical vein cells (EA.hy926). Using the same conditions as with B16-F10, we found that the compound had similar consequences in both human cells as it did in the murine cells treated with less than 50 µM 4-(phenylsulfanyl)butan-2-one. Thus, these results indicated that 4-(phenylsulfanyl)butan-2-one was harmless in three experimental cells.

### 2.3. 4-(Phenylsulfanyl)butan-2 Diminishes Tyrosinase Activity and Melanogenesis in B16-F10 Cells

**Figure 3:**
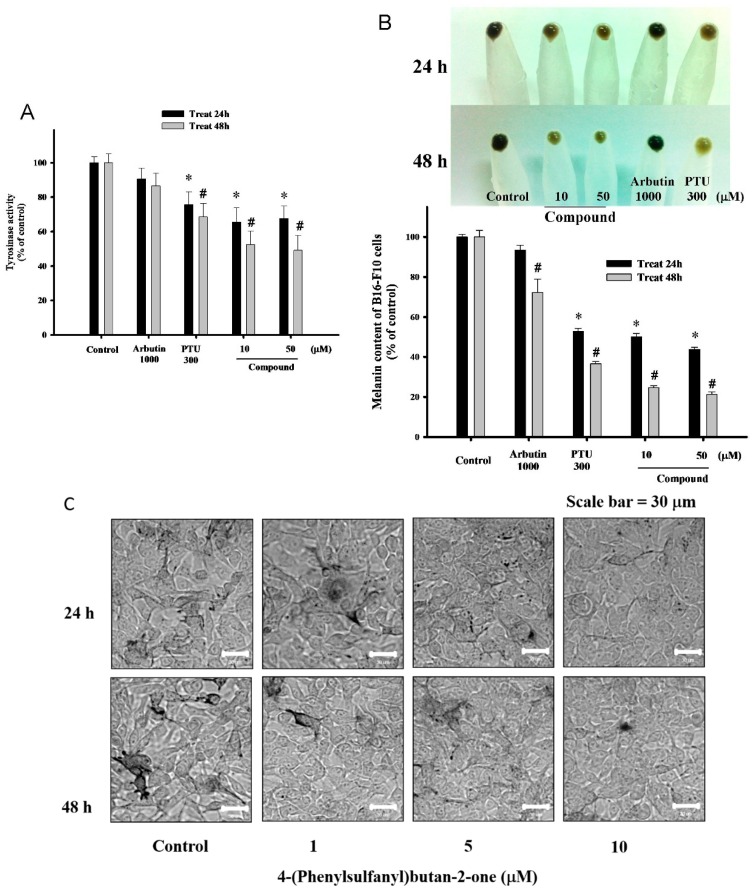
Tyrosinase activity and melanin content of 4-(phenylsulfanyl)butan-2-one treated B16-F10 cells. (**A**) Tyrosinase activities of B16-F10 cells after 24 h treatment with various concentrations of 4-(phenylsulfanyl)butan-2-one; (**B**) Melanin contents and the photographs on the top are from pellet collection; and (**C**) Cultured-cell photographs were taken for melanin accumulation in B16-F10 with various concentration of 4-(phenylsulfanyl)butan-2-one. The vehicle control group is DMSO (0.5%), and arbutin (1000 µM) and PTU (300 µM) are the positive control groups. (* *p* < 0.05 compared to the control group at 24 h; and # *p* < 0.05 compared to the control group at 48 h).

To clearly understand the inhibitory effect of 4-(phenylsulfanyl)butan-2 on melanogenesis, we assessed intracellular tyrosinase activity in B16-F10 cells. Cells were cultured in 10 and 50 µM (suitable concentrations as suggested by the cell viability assay) for two periods of time, 24 h and 48 h. After incubation, tyrosinase activities were suppressed to a greater extent than those of the vehicle control, 1,000 µM arbutin, and 300 µM PTU (Figure 3A). We studied the inhibitory effects of high concentrations of PTU and arbutin, which are renowned melanin inhibitors, on pigment generation in the examination platforms. We further determined the effectiveness of 4-(phenylsulfanyl)butan-2 on melanin production using B16-F10 cells cultured with the same aforementioned agent concentrations. The melanin assay results clearly showed that the sample reduced the melanin content of B16-F10 cells in both time- and dose-dependent manners (Figure 3B,C). 4-(Phenylsulfanyl)butan-2 significantly inhibited melanin synthesis, and produced less than 30% of baseline levels at both doses (10 and 50 µM) for 48 h compared to arbutin or PTU, respectively.

### 2.4. Influences of 4-(Phenylsulfanyl)butan-2-one on Melanin Biosynthesis and Melanosome Maturation in B16-F10 Cells

**Figure 4:**
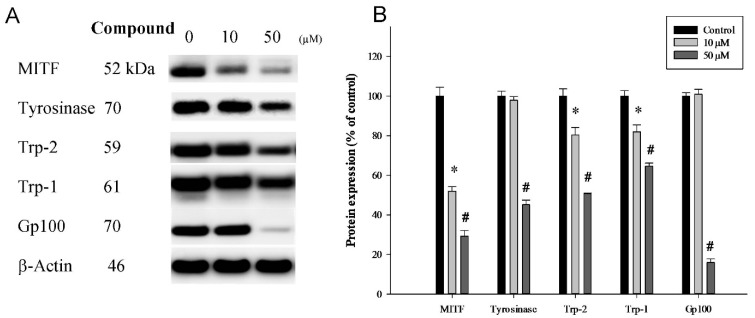
Western blotting was performed to compare melanin related protein expression in B16-F10 cells with and without the addition of 4-(phenylsulfanyl)butan-2-one at concentrations of 10 and 50 μM for 24 h. (**A**) Western blot; and (**B**) Quantification results. (* *p* < 0.05 compared to the control group; and # *p* < 0.05 compared to the 10 µM treatment group).

Melanin biosynthesis takes place in dermal melanocytes, and the substrate amino acid is l-tyrosine. First, l-tyrosine is catalyzed to l-DOPA by tyrosinase, and l-DOPA is converted in to dopaquinone via the same enzyme. Dopaquinone, through catalysis by Trp-2, Trp-1, and tyrosinase, becomes the black or gray pigment, eumelanin; another bypass-path end product is the red or yellow pigment, pheomelanin [7]. We revealed that 4-(phenylsulfanyl)butan-2-one was able to downregulate the protein expression of melanogenesis-related proteins including MITF, tyrosinase, Trp-2, Trp-1, and Gp100 in a dose-dependent manner (Figure 4A). Evident decreases in properties were observed with treatment, and the quantification analysis is shown in Figure 4B.

### 2.5. 4-(Phenylsulfanyl)butan-2-one Effects in An in Vivo Zebrafish Model

**Figure 5:**
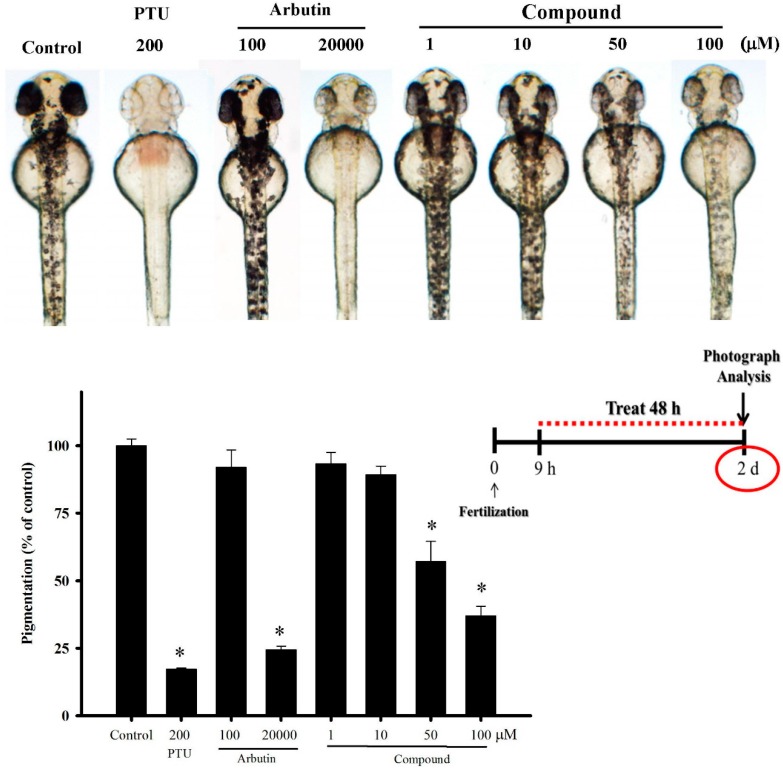
Photos and quantified results of 4-(phenylsulfanyl)butan-2-one on the pigmentation levels of the zebrafish system. PTU (200 µM) and arbutin (100 and 20,000 µM) were the positive control groups. Test compounds were dissolved in 1.0% DMSO, and then added to the embryo medium from 9 to 57 hpf (total 48 h exposure) at 28.5 °C. (* *p* < 0.01, compared to the control group).

Following the *in vitro* tyrosinase inhibition screening, we executed an *in vivo* zebrafish assay to examine the bioactivity of our target compound. All samples and doses being examined consisted of two embryos within one well (on a 96-well plate). The phenotypic effectiveness on zebrafish melanin was observed by analyzing the body pigment under a stereomicroscope at 28.5 °C (Figure 5). The positive control groups were 200 µM PTU, and 100 and 20,000 µM arbutin. Controls and samples were dissolved in 1% DMSO culture medium. We monitored embryo development from 9 to 57 hpf (hours post-fertilization, total two day exposures). Using various concentrations of compound, we identified the appropriate doses for pigment reduction on the exterior surface of zebrafish. After incubation with low doses (1 and 10 µM) of 4-(phenylsulfanyl)butan-2-one, surface melanin was not significantly reduced. Surface melanin diminished about 40% to 60%, in zebrafish incubated with 50 and 100 µM of the compound. Our target compound demonstrated greater apparent de-pigmenting at concentrations higher than 50 µM after 48 h treatments.

**Figure 6:**
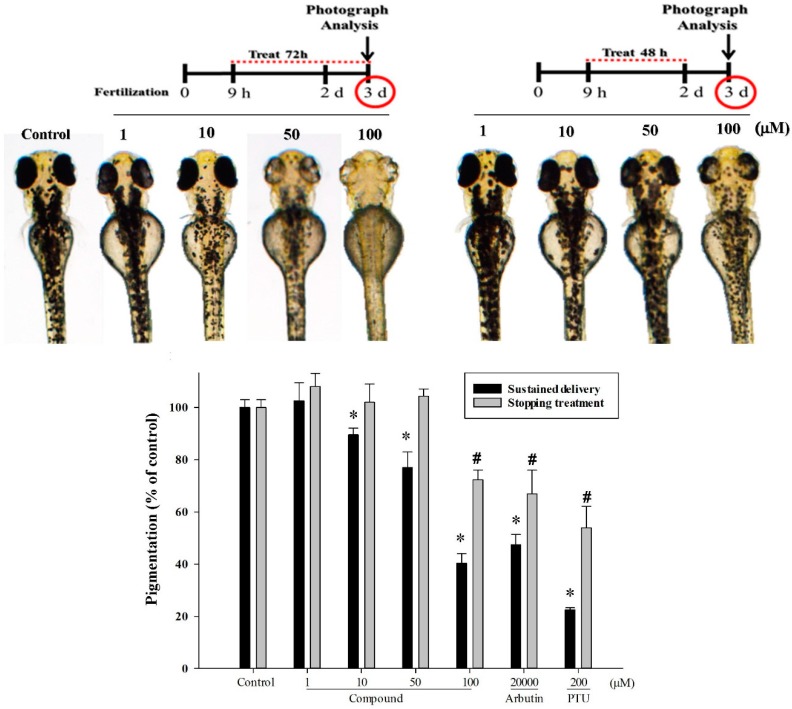
Photoes and quantitated results of 4-(phenylsulfanyl)butan-2-one on the pigmentation levels of the zebrafish system. The experimental conditions were similar to those in Figure 5, except one group had sustained treatment for another 24 h. Phenotype-based evaluations of the zebrafish body pigmentation were then carried out at three dpf. (* *p* < 0.05 compared to the sustained delivery in control group; and # *p* < 0.05 compared to the stopping treatment in control group).

Next, we scrutinized the compound intermittent-treatment response in zebrafish as shown in Figure 6. Briefly, after a 9-h fertilization period and 48 h of treatment, one group was continuously cultured in sample conditions for 24 h, and the other group was without 4-(phenylsulfanyl)butan-2-one in the otherwise same conditions. The compound sustained handling group showed obvious suppression of zebrafish melanin generation at each concentration (10, 50, and 100 µM). On the other side, the compound treatment stopped group presented pigment recovery, and at doses lower than 100 µM, the recovery-pigment contents were similar to the vehicle control group.

**Figure 7:**
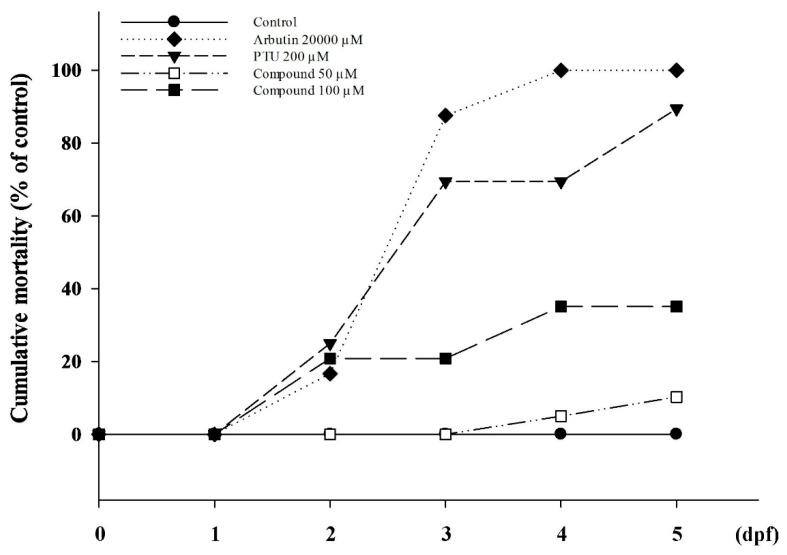
Zebrafish survival rates. Zebrafish were incubated with 4-(phenylsulfanyl)butan-2-one at 50 and 100 µM. Arbutin (20,000 µM) and PTU (200 µM) were used as the positive control groups. All culture media were replaced containing fresh compounds every day.

Finally, we examined zebrafish mortality in 4-(phenylsulfanyl)butan-2-one condition treatments (Figure 7). Culture conditions were essentially the same as those in the general experimental protocols and embryo numbers were increased to 30 for each test. The vehicle control group showed a favorable survival rate until day five. Both positive control groups, arbutin and PTU, presented high death ratios from 72 h onwards, and arbutin at 20,000 µM caused all 30 embryos to die. The experimental groups of 50 and 100 µM 4-(phenylsulfanyl)butan-2-one showed good survival percentages. Additionally, we did not observe any strange behavior or extraordinary mutation in zebrafish in response to the treatment.

## 3. Discussion

The most important pigment determining human skin color is melanin. Melanogenesis suppressors are useful as therapeutics for hyper-pigmentation and have application in the cosmetic industry [7,18,19,20,21,22,23,24,25,26]. The purpose of this study was to identify novel natural and potent inhibitors of melanin production. As initially stated, the inhibitory effect of 4-(phenylsulfanyl)butan-2-one on mushroom tyrosinase inhibition (*in vitro*) was determined. Safe and effective skin lightening compounds are important for preventing hyper-pigmentation. Our target marine derivate compound exhibited diminished mushroom tyrosinase activity (Table 1). Moreover, these results were substantiated in more advanced testing platforms, including mammalian B16-F10 dermal cells and *in vivo* examination models. We further investigated the mushroom tyrosinase inhibitory kinetic parameters of 4-(phenylsulfanyl)butan-2-one. According to the Lineweaver–Burk plot we obtained, the compound was a non-competitive inhibitor of the enzyme substrate.

Suppressed tyrosinase activity has been reported in numerous papers; however, most only tested this in a mushroom enzyme activity platform [27,28,29,30,31]. We acknowledge that mammalian and mushroom tyrosinase catalyses have different reaction mechanisms. Melanin production by the two types of tyrosinases have various hydrophilic water solubility possessions in l-DOPA as the substrate, and l-DOPA stimulates the mushroom tyrosinase but does not affect mammalian tyrosinase activities. In the second stage of testing, we determined whether cellular melanin genesis is suppressed in mammal B16-F10 cells. Using a microscope, we obtained cultured-cell photographs with the melanin accumulations in B16-F10 cells treated with various concentrations of 4-(phenylsulfanyl)butan-2-one. This implies that the compound is a stronger inhibitor than arbutin or PTU. In this mouse epidermal melanoma model, we found that the pigment concentration and tyrosinase activity were significantly reduced by the presence of 4-(phenylsulfanyl)butan-2-one in a dose-dependent manner. Since kojic acid and arbutin are weak epidermal de-pigment agents, and their use at high concentrations causes carcinogenic diseases, our compound was scrutinized for high skin-lightening effectiveness at low concentrations.

In melanin biosynthesis, tyrosinase has a unique influence, but other proteins also contribute to this process, including MITF, Trp-1, Trp-2, and Gp100. DOPA-chrome is catalyzed to 5,6-dihydroxyindole-2-carboxylic acid by Trp-2, and the meddle-product is catalyzed to indole-5,6-quinone carboxylic acid, which is then used to synthesize eumelanin [7]. In our cellular model, 4-(phenylsulfanyl)butan-2-one was shown to downregulate B16-F10 tyrosinase activity. Additionally, MITF, tyrosinase, Trp-1, Trp-2, and Gp100 were significantly and dose-dependently reduced compared to the vehicle control group at 10 and 50 µM. MITF, tyrosinase, Trp-1, and Trp-2 are melanin biosynthesis-related proteins. Gp100 is an epidermal melanocyte lineage-specific protein that is 661 amino acids long, and is a type I trans-membrane glycoprotein enriched within melanosomes [32,33,34]. Melanosomes are the melanin-producing organelles within melanocytes, and Gp100 is involved in melanosome maturation. In future studies we will focus on melanin maturation, transportation, accumulation, and degradation mechanisms.

As a melanin synthesis regulatory agent is being developed, possible harmfulness and reliability must be put into scientists’ considerations, such as the toxicity, allergy, and sensitivity tests, even if most are not confirmed right now. When our dermal pigment inhibitory agent is being considered for potential cosmetic or therapeutic use in humans, the cytotoxic properties of 4-(phenylsulfanyl)butan-2-one become extremely important. There are several famous pigment production suppressors, including kojic acid, arbutin, PTU, and hydroquinone. They are being developed internationally as ingredients in cosmetics at the present time. However, these pigment synthesis suppressors may be tumorigenic at a high dose or with frequent usage [35,36]. Every country has its own laws regarding concentration limits for the use of kojic acid, PTU, arbutin, and other cosmetic additives. In addition to the *in vitro* screening using the mushroom model and mammal cell culture system, an *in vivo* zebrafish model was used as a more physiologically relevant platform to elucidate the mechanism for suppression [37,38,39,40]. The zebrafish system has many advantages: Convenience in observing melanin development, inducible spawning by light, a large quantity of embryos in vertebrates, a rapid melanin synthesis process, and high permeability to small molecules. Moreover, within this system, the agent toxicity is verified simultaneously with effectiveness in a vertebrate system, owing to its similarity to humans in organ systems and gene sequence [41]. 4-(Phenylsulfanyl)butan-2-one demonstrated a noteworthy suppression of zebrafish melanin generation. Furthermore, the compound inhibited melanogenesis even at low concentrations, without apparent severe toxicity to zebrafish. Our results indicated that this whitening substance is safe and effective and that it could be used to prevent hyper-pigmentation.

## 4. Materials and Methods

### 4.1. 4-(Phenylsulfanyl)butan-2-one Chemistry Synthesis Reaction

**Scheme 1:**
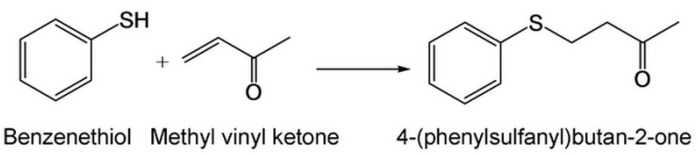
The compound structure and chemical synthesis reaction scheme of 4-(phenylsulfanyl)butan-2-one.

We followed our published papers with minor modification to obtain the target compound [6]. We found that 4-(phenylsulfanyl)butan-2-one had an excellent ability to prevent hyper-pigmentation and whiten skin. Briefly, benzenethiol (2.00 g, 98%, 17.8 mmol) and triethylamine (0.25 mL, 1.78 mmol) were added to a round bottom flask containing 5.0 mL acetone. Followed by stirring at 0 °C, a solution of methyl vinyl ketone (1.38 mL, 90%, 17.8 mmol) in 4.0 mL acetone was slowly added into the mixture. The temperature of the mixture was raised to room temperature and the reaction was continued for 16 h. The solvent free product was subject to silica gel column chromatography, eluted with n-hexane/ethyl acetate (25:1), to yield 4-(phenylsulfanyl)butan-2-one (2.80 g, yield 87%). The reaction is shown in Scheme 1.

### 4.2. Tyrosine Activity Measurement (in Vitro Mushroom Model)

Mushroom tyrosinase activity inhibition was determined spectrophotometrically (Bio-Rad 3550; Bio-Rad Laboratories, Inc.: Hercules, CA, USA), according to the method described previously with minor modifications [27]. Evaluations were conducted in a 96-well plate at 490 nm, and all substances were dissolved in dimethyl sulfoxide (DMSO). Arbutin (1,000 µM) and PTU (300 µM) were used as a positive control in the tyrosinase inhibitory assay. Samples were incubated with 25 U/mL mushroom tyrosinase, and l-tyrosine (2 mM) in phosphate buffer (pH 6.8) was added at 37 °C for 30 min. Tyrosinase inhibitory activity was determined by the following equation:

(1) $$\text{Tyrosinase inhibitory activity }\left(\text{\%}\right)=\;\frac{\left[\left(A\;-B\right)\;-\left(C\;-D\right)\right]}{\left(A\;-B\right)}\;\times \;100\text{\%}$$

where *A* is the optical density (OD_490_) without the testing substance; *B* is the OD_490_ without the testing substance, but with tyrosinase; *C* is the OD_490_ with the testing substance; and *D* is the OD_490_ with the testing substance, but without tyrosinase.

### 4.3. Cell Viability Assay

The murine melanoma cells (B16-F10), human foreskin fibroblasts (Hs68), and human umbilical vein cell line (EA.hy926) were separately cultured on 96-well plates [28]. B16-F10 cells (No. CRL-6475) were obtained from the American Type Culture Collection (ATCC: Manassas, VA, USA); Hs68 cells (No. CRL-1635) were purchased from ATCC. Hs68 is one of a series of human foreskin fibroblast lines developed at the Naval Biosciences Laboratory (NBL, Oakland, CA, USA); and EA.hy926 cells (No. CRL-2922) were also acquired from ATCC. The human umbilical vein cell line, EA.hy926, was established by fusing primary human umbilical vein cells with a thioguanine-resistant clone of A549 cell (human lung cancer) by exposure to polyethylene glycol. The testing compound at suitable doses was added to each of the three cell cultures. The cells were cultured in DMEM medium within a 5% CO_2_ atmosphere humidified incubator at 37 °C for 24 h. After 24 h incubation, 10% alamarBlue^®^ (Biosource, CA, USA) was aseptically added to measure cell viability, according to commercial kit protocols. The vehicle control group (without 4-(phenylsulfanyl)butan-2-one) was defined as 100%. The optical absorbance values (*A*) of the supernatants were quantified at 570 nm, and the cell viabilities were analyzed according to the following formula:

(2) $$\text{Cell viability }\left(\text{\%}\right)=\;\frac{\left(A_{\text{sample}}\;-A_{\text{blank}}\right)}{\left(A_{\text{control}}\;-A_{\text{blank}}\right)}\;\times \;100\text{\%}$$

### 4.4. Assay on Cellular Tyrosinase Activity (Cell-Based Evaluation)

Tyrosinase activity was estimated by measuring the rate of dopachrome formation, based on the method described previously with minor modifications [42]. B16-F10 cells (10^5^ per well) were placed in 24-well plates in 500 µL DMEM medium with 72×10^−3^ mg/mL l-tyrosine that acts as not only substrate but also regulator of melanogenesis [43,44], and then various concentrations of testing samples were added to the medium and incubated for 2 days. The sample-treated cells were washed with phosphate-buffered saline (PBS) and lysed with 1% Triton X-100/PBS. The enzyme extract of the cellular lysate was added to 10 µL 2 mM l-tyrosine as substrates mixed in 0.1 M phosphate buffer (pH 6.8). This reaction was then incubated at 37 °C for 3 h in the dark, and the absorbance at 475 nm was measured on a spectrophotometer. The vehicle control group tyrosinase activity (without 4-(phenylsulfanyl)butan-2-one) was defined as 100%.

### 4.5. Melanin Content Assay

According to a previous method with minor modifications [18,45], cell pellets were dissolved in 1.0 N NaOH containing 10% DMSO, heated at 80 °C for 1 h, uniformly mixed in the solution, and then the absorbance at 490 nm was measured via a spectrophotometer. As above, the vehicle control group of melanin content was defined as 100%.

### 4.6. Western Blot

B16-F10 cells were treated with 4-(phenylsulfanyl)butan-2-one or a vehicle control for 24 h. Cells were washed twice with ice cold PBS buffer, harvested, and disrupted in lysis buffer (2% SDS, 10% glycerol, 0.1% bromophenol blue, 2% 2-mercaptoethanol, and 50 mM Tris-HCl; pH 7.2) [3,5]. The cell lysate was centrifuged at 12,000 × *g* for 20 min at −4 °C, and the supernatant concentration was determined using the DC protein assay kit (Bio-Rad: Hercules, CA, USA). In total, 40 µg of protein was separated via 7% sodium dodecyl sulfate polyacrylamide gel electrophoresis (SDS-PAGE) and blotted onto polyvinylidene difluoride membranes (PVDF, Millipore: Bedford, MA, USA). The membranes were blocked with 5% non-fat skim milk in a tris-buffered saline (TBS)-Tween 20 solution. Tyrosinase (Abcam: ab52493, 1:1000, Cambridge, UK), Trp-1 (Abcam, ab83774, 1:1000), Trp-2 (Abcam, ab74073, 1:1000), Gp100 (Abcam, ab52058, 1:250), MITF (Abcam, ab20663, 1:1000) and β-actin (Sigma aldrich, A5316, 1:2000, USA) were all detected with a rabbit polyclonal anti-tyrosinase antibody, a rabbit polyclonal anti-Trp-1 antibody, a rabbit polyclonal anti-Trp-2 antibody, a goat monoclonal anti-Gp100 antibody, and a mouse monoclonal anti-β-actin antibody (Abcam PLC: Cambridge, UK). The PVDF films were further incubated with horseradish peroxidase-conjugated secondary antibody. Immunoreactive bands were visualized by enhanced chemiluminescence. All bound antibodies were then detected with a BioSpectrum^®^ Imaging System (UVP: LLC, Upland, CA, USA).

### 4.7. Zebrafish (in Vivo Assay)

Synchronized embryos were collected and arrayed by pipette, three embryos per well, in a 96-well plate containing 200 µL embryo medium, according to a previous method [45]. Test compounds were dissolved in 1% DMSO and then added to the embryo medium from 9 to 57 hpf (total 48 h exposure) at 28.5 °C. The positive controls were 200 µM PTU and 20,000 µM arbutin, respectively. The effects on zebrafish pigmentation were observed under a stereomicroscope. Phenotype-based evaluations of body pigmentation were then carried out at 57 hpf. For observation, embryos were dechorionated by forceps, anesthetized in tricaine methanesulfonate solution (Sigma-Aldrich Co.: St. Louis, MO, USA), mounted in 1% methyl cellulose on a depression slide (Aquatic Eco-Systems: Apopka, FL, USA), and photographed under a stereomicroscope Z16 (Leica Microsystems: Wetzlar, Germany). Images were captured using a SPOT CCD Idea integrating camera (Diagnostic Instruments Inc.: Sterling Heights, MI, USA). Afterwards, images were taken using the Scion Image alpha 4.0.3.2 software (Scion Co.: Torrance, CA, USA) by a blinded observer. The pixel measurement analyzer program was then used to count the area of the zebrafish image pigmentation. The quantification of pigmentation data was expressed as a percent change compared to the control group, which was considered 100%.

### 4.8. Statistical Analysis

Results are presented as a mean value of the data obtained from triplicate experiments. Student’s *t*-test was used to determine the level of significance.

## 5. Conclusions

In this study, we developed the anti-melanogenesis abilities of our target compound, 4-(phenylsulfanyl)butan-2-one. Via the mushroom tyrosinase inhibition test, we found that the compound had the tyrosinase inhibitory capacity, and the kinetic analysis showed that the suppressive mechanism was noncompetitive. In murine melanoma B16-F10 cells, 4-(phenylsulfanyl)butan-2-one also inhibited the cell-based tyrosinase activity, and decreased the melanin production. To confirm the pathway of melanogenesis inhibition, we used the western blotting to prove that MITF, tyrosinase, Trp-1 and Trp-2 were inhibited by 4-(phenylsulfanyl)butan-2-one, and the compound arrested the melanosome mature by Gp100 repression. Further, we executed the *in vivo* zebrafish model to determine melanin generation diminish by our compound. In B16-F10, Hs68 and EA.hy926 cell viability, and zebrafish models, it did not showed the cytotoxic, carcinogenic or teratogenic properties. We suggested that the 4-(phenylsulfanyl)butan-2-one is safe and efficacious to be developed into a skin whitening substance.
